# Substrate size-dependent conformational changes of bacterial pectin-binding protein crucial for chemotaxis and assimilation

**DOI:** 10.1038/s41598-022-16540-5

**Published:** 2022-07-25

**Authors:** Kotaro Anamizu, Ryuichi Takase, Mamoru Hio, Daisuke Watanabe, Bunzo Mikami, Wataru Hashimoto

**Affiliations:** 1 Laboratory of Basic and Applied Molecular Biotechnology, Division of Food Science and Biotechnology, Graduate School of Agriculture, Kyoto University, Uji, Kyoto, 611-0011 Japan; 2 Laboratory of Basic and Applied Molecular Biotechnology, Department of Food Science and Biotechnology, Faculty of Agriculture, Kyoto University, Uji, Kyoto, 611-0011 Japan; 3 Laboratory of Metabolic Sciences of Forest Plants and Microorganisms, Research Institute for Sustainable Humanosphere, Kyoto University, Uji, Kyoto, 611-0011 Japan; 4 Laboratory of Structural Energy Bioscience, Institute of Advanced Energy, Kyoto University, Uji, Kyoto 611-0011 Japan

**Keywords:** Bacteriology, X-ray crystallography

## Abstract

Gram-negative *Sphingomonas* sp. strain A1 exhibits positive chemotaxis toward acidic polysaccharide pectin. SPH1118 has been identified as a pectin-binding protein involved in both pectin chemotaxis and assimilation. Here we show tertiary structures of SPH1118 with six different conformations as determined by X-ray crystallography. SPH1118 consisted of two domains with a large cleft between the domains and substrates bound to positively charged and aromatic residues in the cleft through hydrogen bond and stacking interactions. Substrate-free SPH1118 adopted three different conformations in the open form. On the other hand, the two domains were closed in substrate-bound form and the domain closure ratio was changed in response to the substrate size, suggesting that the conformational change upon binding to the substrate triggered the expression of pectin chemotaxis and assimilation. This study first clarified that the solute-binding protein with dual functions recognized the substrate through flexible conformational changes in response to the substrate size.

## Introduction

Motile bacteria sense concentration gradients of surrounding chemicals and move toward environments with favorable nutritional conditions, while moving away from toxic materials. This type of bacterial motility is called chemotaxis^1^. Motile bacteria have flagellum consisting of a basal body, hook, and filament, which regulate the direction of movement by controlling the direction of rotation of the flagellum^2^. For example, *Escherichia coli* moves forward by rotating the flagellum counter-clockwise and changes the direction by rotating it clockwise^3^. The expression of bacterial chemotaxis relies on methyl-accepting chemotaxis protein (MCP) and Che proteins. MCP is regulated by methylation and demethylation^4^. Once MCPs recognize chemotactic substrates, signals are generated and transmitted through the cell membrane into the cytoplasm. The signal activates the Che protein in the cytoplasm, which regulates the direction of flagellar rotation through a two-component regulatory system, resulting in chemotaxis^5^.

*Sphingomonas* sp. strain A1 isolated from the soil is a gram-negative bacterium and grows well on alginate and pectin. Alginate is an acidic polysaccharide consisting of a linear chain of β-d-mannuronic acid and α-l-guluronic acid and is a major carbohydrate in brown seaweed^6^. Pectin, which is abundant in plant cell walls, is a heteropolysaccharide composed of three regions: polygalacturonan (PG), rhamnogalacturonan type I (RG-I), and rhamnogalacturonan type II (RG-II)^7^. PG is the major constituent region of pectin and consists of a linear polymerization of d-galacturonic acid (GalUA) with α-1,4 linkages. Some of the GalUA residues in PG are methyl esterified. RG-I is a heteropolysaccharide consisting of a main chain composed of repeating α-1,4 and α-1,2 linkages of GalUA and l-rhamnose (Rha) with three side chains of arabinan, galactan, and arabinogalactan^8^. RG-II has PG as the main chain and various sugars, including rare sugars such as apiose and aceric acid as the side chains^9^.

In contrast to other polysaccharide-assimilating bacteria, strain A1 is peculiar in that strain A1 cells incorporate alginate as a polysaccharide into the cytoplasm and degrade the polymer^10^. Alginate is incorporated into the periplasm through the mouth-like pit formed on the cell surface of strain A1^11^. The alginate-binding proteins (AlgQ1 and AlgQ2) localized in the periplasm transfer alginate to the ATP-binding cassette (ABC) transporter (AlgM1M2SS) in the inner membrane. Alginate is transported directly into the cytoplasm^12,13^ and finally degraded in the cytoplasm by endo- and exo-type alginate lyases (A1-I, II, III, and IV) into unsaturated monosaccharides^14,15^. The unsaturated monosaccharides are converted to glyceraldehyde-3-phosphate and pyruvate through reactions of reductase (A1-R, A1-R'), kinase (A1-K), and aldolase (A1-A)^16,17^.

There are two derivatives of strain A1 (strains A1-M5 and A1-MP) showing chemotaxis using flagellum toward acidic polysaccharide(s)^18,19^. They were isolated by subculturing the non-motile strain A1 wild-type on a soft agar plate. This is the first example of bacterial chemotaxis against macromolecules. Strain A1-M5 shows chemotaxis toward alginate, but not toward pectin^20^. On the other hand, strain A1-MP exhibits chemotaxis toward both alginate and pectin^19^. SPH1118 has been identified as the essential protein for chemotaxis toward pectin through genome comparison, gene disruption, and complementation experiments^19^. SPH1118 shows high homology with the solute-binding protein (SBP) associated with the bacterial ABC transporter. The molecular weight of SPH1118 is 69 kDa, which is large among SBPs. In addition, SPH1118 deficient strain A1-MP (strain *Δsph1118*) not only lacked pectin chemotaxis but also decreased pectin-assimilating ability^19^. The recombinant protein was produced by constructing the expression system for SPH1118 in *E. coli* to evaluate its binding ability to pectin. The purified SPH1118 shows a high affinity with pectin [dissociation constant (*K*_d_) = 9.28 ± 1.12 μM]^19^.

Some SBPs are known to function as receptors for chemotaxis in the same way as SPH1118. The gram-negative bacterium *Dickeya dadantii* (formerly known as *Erwinia chrysanthemi*) has an SBP called TogB to exhibit chemotaxis toward pectin oligosaccharides. TogB has two functions: it acts as an SBP that conveys pectin oligosaccharides to the ABC transporter and also functions as a receptor for chemotaxis^21^. Another well-known example is the maltose-binding protein (MBP) from *E. coli*. The MBP not only functions as an SBP to deliver maltose to the ABC transporter but also interacts with the periplasmic region of Tar, the aspartate chemotaxis receptor, and transmits chemotactic signals^22–24^. However, as far as we know, there have been no reports on SBPs triggering chemotaxis toward polysaccharides. This study deals with the mechanism of polysaccharide recognition of SPH1118 through flexible conformational changes in response to the molecular weight of the substrate.

## Results

### SPH1118 can recognize oligosaccharides and monosaccharides as well as polysaccharides

SPH1118 exhibits a high affinity with polysaccharides such as pectin, PG, and RG-I at the μM level^19^. SPH1118 hardly binds to digalacturonic acid and GalUA at 1.5 mM, suggesting that SPH1118 prefers to recognize polysaccharides. Its affinity with oligosaccharides or monosaccharides at high concentrations has been unknown. SPH1118 was expressed by the recombinant *E. coli* and purified to homogeneity to investigate the binding properties of SPH1118 to oligosaccharides and monosaccharides. As previously reported^19^, the purified SPH1118 was eluted as a single peak by gel filtration column chromatography (Supplementary Fig. 1a). It was subjected to sodium dodecyl sulfate–polyacrylamide gel electrophoresis (SDS-PAGE) to confirm its high purity (Supplementary Fig. 1b). The main chain of RG-I was prepared by acid hydrolysis of RG-I to obtain pectin oligosaccharide. The main chain of RG-I was then degraded by the exo-type lyase YesX, and reaction products were confirmed by thin-layer chromatography (TLC) (Supplementary Fig. 1c). X-ray crystallography of the SPH1118-substrate complex later described revealed that the pectin oligosaccharide was an unsaturated trigalacturonic acid (ΔtriGalUA) whose non-reducing end was unsaturated d-galacturonic acid (ΔGalUA).

**Figure 1:**
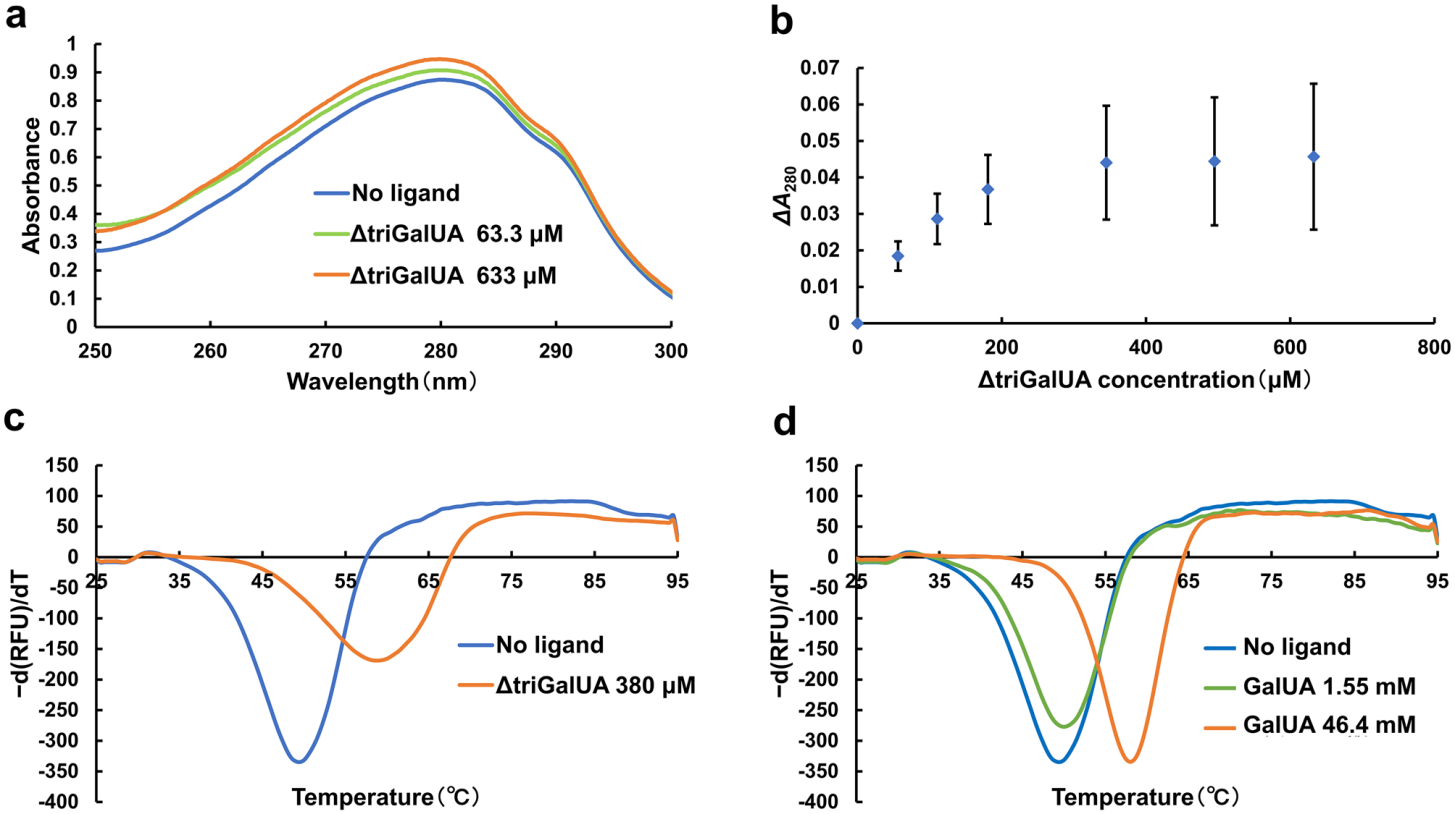
Binding assay of SPH1118 with ΔtriGalUA and GalUA. (**a**) The UV spectra of SPH1118 in the absence (blue) and presence of 63.3 μM (green) and 633 μM ΔtriGalUA (orange). (**b**) Differential absorbance at 280 nm (*ΔA*_280_) as a function of ΔtriGalUA concentration. Each data point represents the mean ± standard error (SE) from three independent experiments. (**c**) The negative derivative curve plots obtained from the fluorescence profiles of SPH1118 in the absence (blue) and presence of 380 μM ΔtriGalUA (orange). (**d**) The negative derivative curve plots obtained from the fluorescence profiles of SPH1118 in the absence (blue) and presence of 1.55 mM (green) and 46.4 mM GalUA (orange).

The affinity of SPH1118 with ΔtriGalUA was examined through UV absorption spectra assay. This method evaluates the affinity with the substrate by measuring the change in the UV absorption spectra upon binding to the substrate. The UV absorption spectra varied in a ∆triGalUA concentration-dependent manner (Fig. 1a). In addition, absorbance at 280 nm (*A*_280_) in the presence of various concentrations of ΔtriGalUA was measured. As a result, the absorbance increased as the concentration of ΔtriGalUA increased (Fig. 1b). *K*_d_ value of SPH1118 for ΔtriGalUA was calculated to be 96.2 ± 10.4 μM (Supplementary Table 1 by approximating the obtained saturation curve to the Langmuir equation^25^. The binding of SPH1118 to ΔtriGalUA was also evaluated by differential scanning fluorimetry (DSF). This assay evaluates the binding of the protein to the ligand based on the increase of the thermal stability upon binding to the ligand. The thermal stability is evaluated by the melting temperature (*T*_m_) corresponding to the inflection point of the fluorescence profile. Generally, protein–ligand binding shifts *T*_m_ value higher^26^. *T*_m_ values in the absence and presence of 380 μM ΔtriGalUA were determined as 49.5 and 58.8 °C, respectively. The thermal stability was increased in the presence of ΔtriGalUA at 380 μM (Fig. 1c). Therefore, consistent with the results of UV absorption spectra, SPH1118 was found to bind to ΔtriGalUA, although the *K*_d_ value was remarkably higher than that toward pectin (*K*_d_ = 9.28 ± 1.12 μM). DSF also analyzed the binding of SPH1118 to GalUA at high concentrations. *T*_m_ values in the absence of GalUA, in the presence of 1.55 mM, and 46.4 mM GalUA were 49.5 °C, 50.0 °C, and 57.8 °C, respectively, showing the increase of the thermal stability in the presence of GalUA at 46.4 mM (Fig. 1d). This suggested that SPH1118 could also bind to GalUA at high concentration.

### Crystal structure of SPH1118

**Figure 2:**
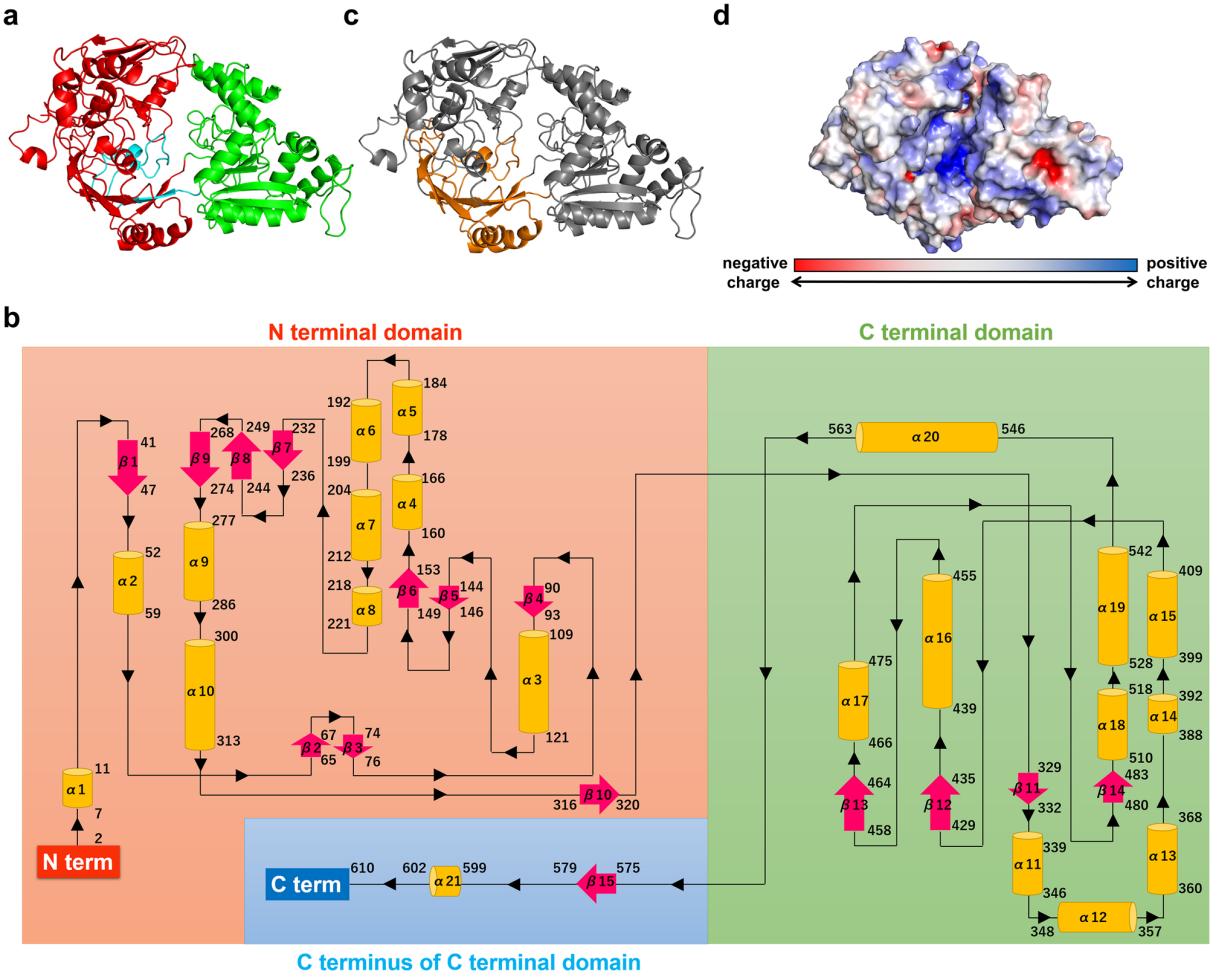
The crystal structure and surface charge of SPH1118. (**a**) Overall structure of substrate-free SPH1118 (open form). Red, N terminal domain (residues 2–322); green, C terminal domain (residues 323–572); cyan, C terminus of C terminal domain (residues 573–610). (**b**) Topology diagram of SPH1118. Orange, α-helix; magenta, β-strand; red, N terminal domain; green, C terminal domain; cyan, C terminus of C terminal domain. (**c**) The extra domain of SPH1118 (open form). Orange, extra domain (residues 41–47, 232–320, and 573–610); gray, other residues. (**d**) Surface charge profile of SPH1118 at pH 7.0. Blue and red surfaces refer to positively and negatively charged areas, respectively.

Crystals were prepared under various conditions using purified SPH1118 with 619 residues, including C terminal 6 × His residues, to determine the crystal structure of SPH1118 (open form, PDB-ID: 7VEQ) at 1.7 Å resolution by X-ray crystallography (Fig. 2a). Note that the 26th alanine was defined as Ala1 because N terminal signal sequence consisting of 25 residues was removed, as reported previously^19^. Data collection and refinement statistics are shown in Table 1. The space group of this crystal was *C*222_1_. The lattice constants of the unit were *a* = 50.9 Å, *b* = 151.7 Å, *c* = 179.5 Å. One molecule was included in the asymmetric unit of the crystal. SPH1118 was found to be composed of two α/β domains: N terminal domain (residues 2–322, Fig. 2a red) and C terminal domain (residues 323–572, Fig. 2a green). Residues 1 and 611–619 were unable to be assigned because of the poor electron density map. There was a cleft considered to be a substrate-binding site between the two domains. In general, SBPs associated with ABC transporters consist of two domains^27^, also seen in SPH1118. Two loops connected the two domains of SPH1118. SPH1118 consisted of 21 α-helices and 5 β-sheets built from 15 β-strands (Fig. 2b). The C terminus of the C terminal domain (residues 573–610, Fig. 2a cyan) in SPH1118 formed a small additional domain and was located in the direction of the N terminal domain. SBPs are classified into seven clusters (A–G) based on their structural features^28^. SPH1118 was categorized into cluster C because of the structural features. SPH1118 had a large molecular weight of 69 kDa and an extra domain (residues 41–47, 232–320, and 573–610) which is characteristic of SBPs in cluster C (Fig. 2c). SBPs classified as cluster C have been reported to bind to a wide variety of substrates including oligopeptides, arginine, nickel ions, and cellobiose^28^. SPH1118 is the first example of SBP in cluster C that binds to polysaccharide such as pectin. The surface electrostatic potentials of SPH1118 calculated at pH 7.0 showed that the cleft between the two domains was positively charged (Fig. 2d).

**Table 1:** Data collection and refinement statistics.

	SPH1118 (open form)	SPH1118 (full open form)	SPH1118 (closed form)	SPH1118/GalUA	SPH1118/2 × MES	SPH1118/ΔtriGalUA
PDB-ID	7VEQ	7VER	7VET	7VEU	7VEV	7VEW
Space group	*C*222_1_	*P*2_1_2_1_2_1_	*P*1	*P*2_1_2_1_2_1_	*P*1	*P*2_1_
**Unit cell parameters**						
*a*, *b*, *c* (Å)	50.9, 151.7, 179.5	80.0, 84.7, 90.0	50.6, 54.9, 112.8	72.7, 86.7, 97.3	49.1, 54.2, 57.0	57.7, 83.7, 148.5
*α*, *β*, *γ* (°)			103.0, 97.4, 92.4		75.9, 66.9, 87.9	90.0, 101.0, 90.0
**Data collection**						
Wavelength (Å)	1.00	1.00	1.00	1.00	1.00	1.00
Resolution limit (Å)	48.2–1.70 (1.74–1.70)	48.8–1.70 (1.80–1.70)	48.2–2.25 (2.39–2.25)	42.4–1.74 (1.84–1.74)	50–1.50 (1.53–1.50)	48.6–1.90 (2.02–1.90)
Total reflections	797,534 (34,931)	715,350 (79,004)	155,361 (25,403)	722,190 (116,963)	231,683 (10,578)	625,469 (92,848)
Unique reflections	158,814 (10,681)	66,777 (9726)	53,447 (8527)	63,700 (10,044)	79,704 (3778)	108,404 (17,122)
Redundancy	5.02 (3.27)	10.7 (8.12)	6.12 (6.27)	6.17 (6.34)	2.90 (2.80)	5.77 (5.42)
Completeness (%)	97.8 (91.0)	98.3 (89.8)	95.9 (94.0)	99.3 (98.1)	99.9 (99.1)	99.5 (97.8)
*I*/Sigma (*I*)	11.1 (1.87)	33.3 (6.26)	14.2 (7.03)	25.6 (4.98)	27.4 (6.46)	11.3 (2.13)
*R*_merge_ (%)	8.6 (55.5)	4.5 (25.3)	5.5 (10.9)	6.8 (52.9)	5.5 (13.9)	8.6 (64.5)
CC_1/2_ (%)	99.7 (69.9)	100 (98.1)	99.4 (98.3)	99.9 (95.3)	99.7 (97.4)	99.8 (89.5)
**Refinement**						
Model (residue/water/ligand)	609/472/0	610/555/0	1218/186/0	612/345/1	612/507/2	1218/479/2
Resolution limit (Å)	48.22–1.70 (1.72–1.70)	48.85–1.70 (1.72–1.70)	43.53–2.25 (2.29–2.25)	40.44–1.74 (1.76–1.74)	26.12–1.50 (1.52–1.50)	48.6–1.92 (1.94–1.92)
Used reflections	76,805	66,748	53,446	63,694	79,607	105,649
Completeness (%)	99.5 (92.0)	98.2 (79.0)	96.0 (89.0)	99.3 (96.0)	94.7 (84.0)	99.8 (100)
*R*-factor (%)	16.8 (26.5)	17.6 (20.5)	18.7 (21.7)	15.4 (25.1)	13.9 (13.5)	18.9 (31.8)
*R*_free_ (%)	20.0 (26.4)	21.1 (25.9)	23.7 (29.5)	18.2 (30.0)	18.6 (23.6)	21.9 (33.2)
Clash score	3.24	2.97	2.58	2.62	1.81	3.98
Rotamer outlier (%)	1.13	0.95	1.63	2.06	0.56	1.82
**RMSD**						
Bond (Å)	0.006	0.005	0.008	0.006	0.005	0.007
Angle (°)	0.817	0.793	0.998	0.823	0.829	0.838
**Ramachandran plot (%)**						
Preferred regions	95.4	96.2	95.2	96.2	95.7	95.8
Allowed regions	4.28	3.62	4.53	3.77	4.10	3.79
Outlier regions	0.33	0.16	0.25	0.00	0.16	0.41

Data of the highest shells are given in parentheses.

### SPH1118 changes its conformation in response to the substrate size

Various crystals were prepared (Supplementary Table 2). GalUA, ΔtriGalUA, and PG were used as substrate candidates to determine the structure of SPH1118-substrate complex. Regarding GalUA and PG, sodium GalUA and sodium PG neutralized with NaOH were used to avoid denaturation of SPH1118. The crystal structures of SPH1118 with six different conformations, including SPH1118 (open form), were determined by X-ray crystallography using these substrates under various conditions. Three crystal structures with different conformations were obtained as substrate-free structures [SPH1118 (open form), SPH1118 (full open form, PDB-ID: 7VER), and SPH1118 (closed form, PDB-ID: 7VET)]. The crystallization conditions of SPH1118 (open form), SPH1118 (full open form), and SPH1118 (closed form) contained the substrates (ΔtriGalUA or sodium PG). However, no substrates were bound to SPH1118 in these structures. Three crystal structures in complex with different ligands were also obtained as follows: SPH1118/GalUA (PDB-ID: 7VEU), SPH1118/2 × 2-(*N*-morpholino)ethanesulfonic acid (MES) (PDB-ID: 7VEV), and SPH1118/ΔtriGalUA (PDB-ID: 7VEW), which bound to a GalUA, two molecules of MES, and a ΔtriGalUA, respectively. Two molecules were included in the asymmetric unit of the crystals of SPH1118 (closed form) and SPH1118/ΔtriGalUA. Root mean square deviations (RMSDs) between two monomers in the same crystals were calculated, respectively, indicating that there were few structural differences [SPH1118 (closed form), 0.543 Å; SPH1118/ΔtriGalUA, 0.106 Å]. Although MES is not an intrinsic substrate, the molecule is similar to GalUA in molecular weight, structure, and electrical charge. The molecular weights of GalUA and MES are 194.1 and 195.2, respectively. Both compounds contained six-membered ring structures. The strongly negatively charged sulfo group of MES was considered to correspond to the carboxy group of GalUA. In addition to these similarities, the reservoir solution contained MES at a high concentration (0.1 M) as a buffer component, which probably resulted in the binding of two molecules of MES as substrate analogs. Moreover, the binding of SPH1118 to MES was evaluated by DSF, but little shift in *T*_m_ was observed in the presence of MES at 0.1 M. *T*_m_ values in the absence and presence of 0.1 M MES were 56.2 °C and 55.8 °C, respectively. Therefore, affinity with MES was hardly confirmed in this assay (Supplementary Fig. 2a).

**Figure 3:**
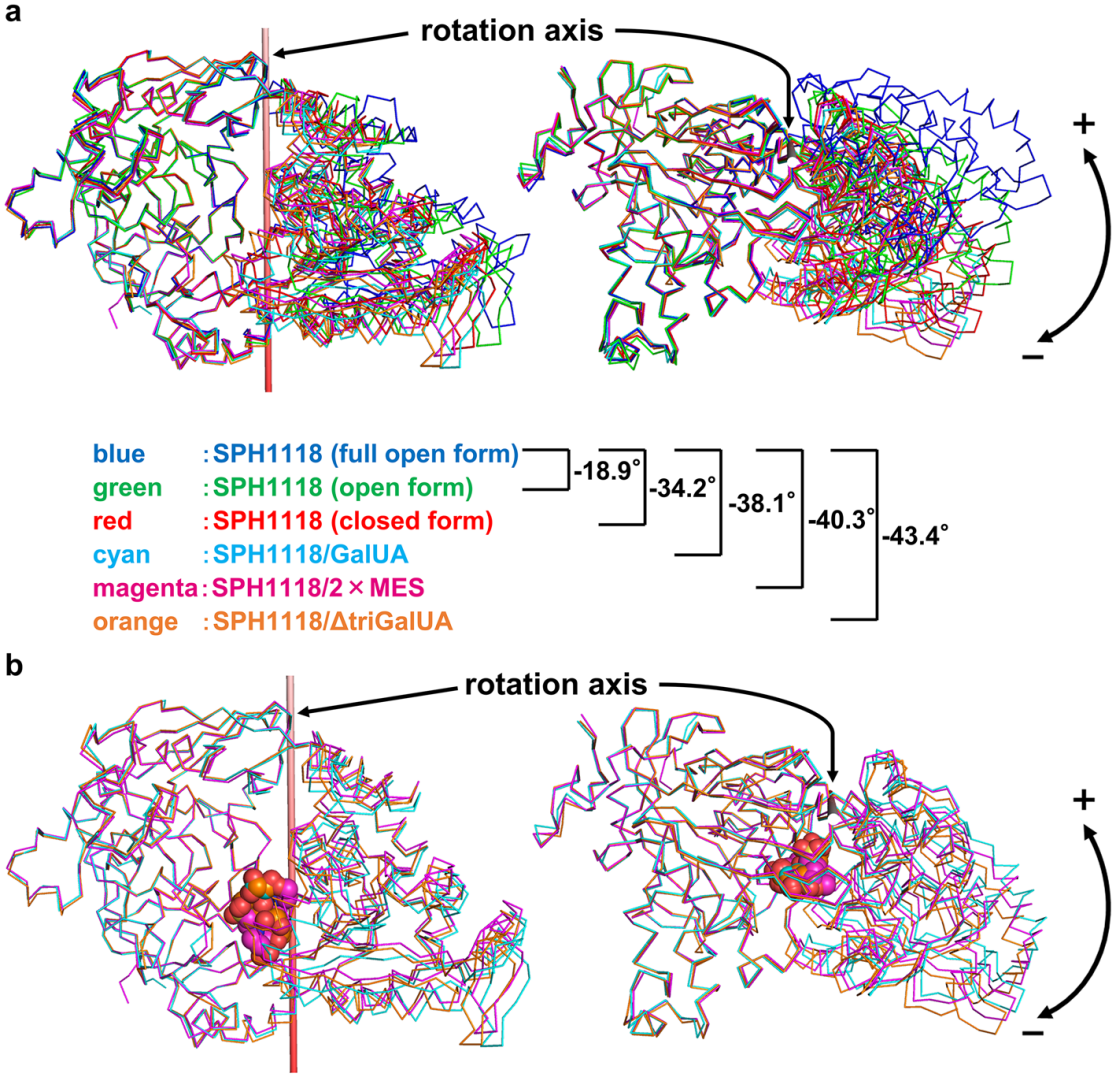
Conformational changes of SPH1118. (**a**) N terminal domains of ligand-free and -bound SPH1118 were superimposed. The right structure is rotated 90° toward the reader relative to the left one. Blue, SPH1118 (full open form); green, SPH1118 (open form); red, SPH1118 (closed form); cyan, SPH1118/GalUA; magenta, SPH1118/2 × MES; orange, SPH1118/ΔtriGalUA. Ribbon models show the main chains of SPH1118. (–) represents the direction in which the C terminal domain closes. Compared with the C terminal domain of SPH1118 (full open form), those of SPH1118 (open form), SPH1118 (closed form), SPH1118/GalUA, SPH1118/2 × MES, and SPH1118/ΔtriGalUA were closed by 18.9°, 34.2°, 38.1°, 40.3°, and 43.4°, respectively. (**b**) N terminal domains of ligand-bound SPH1118 were superimposed. The right structure is rotated 90° toward the reader relative to the left one. Ribbon and ball models show the main chain of SPH1118 and each ligand (GalUA, MES, and ΔtriGalUA), respectively. Cyan, SPH1118/GalUA; magenta, SPH1118/2 × MES; orange, SPH1118/ΔtriGalUA.

Data collection and refinement statistics of X-ray crystallography for each structure are shown in Table 1. All six structures were superimposed based on the Cα in the N terminal domain to compare the differences of angles between N and C terminal domains. The evaluated closure degrees between two domains are shown in Fig. 3a. Compared to the C terminal domain of SPH1118 (full open form), those of SPH1118 (open form) and SPH1118 (closed form) were closed by 18.9° and 34.2°, respectively, suggesting that the domain of SPH1118 was flexibly opened and closed even in the substrate-free state. All complexed conformations were more closed than substrate-free conformations. SPH1118 seemed to recognize the substrate through the Venus fly-trap mechanism generally found in SBPs^29^. Interestingly, the degree of closure also varied among the complexed conformations. Compared to the C terminal domain of SPH1118 (full open form), those of SPH1118/GalUA, SPH1118/2 × MES, and SPH1118/ΔtriGalUA were closed by 38.1°, 40.3°, and 43.4°, respectively. Since the C terminal domain of SPH1118/ΔtriGalUA was more closed than that of SPH1118/GalUA, SPH1118 probably regulated the degree of closure of the domain in response to the molecular weight of the substrate. Regarding the ligand-binding site, each ligand of SPH1118/GalUA, SPH1118/2 × MES, and SPH1118/ΔtriGalUA was accommodated in the same cleft with a positive charge between the two domains (Fig. 3b). Since pectin was negatively charged, SPH1118 probably binds to pectin through the electrical charge interaction. This fact also supported that MES bound to SPH1118 as a substrate analog under the crystallizing conditions.

### Binding mode of SPH1118 to the ligand

**Figure 4:**
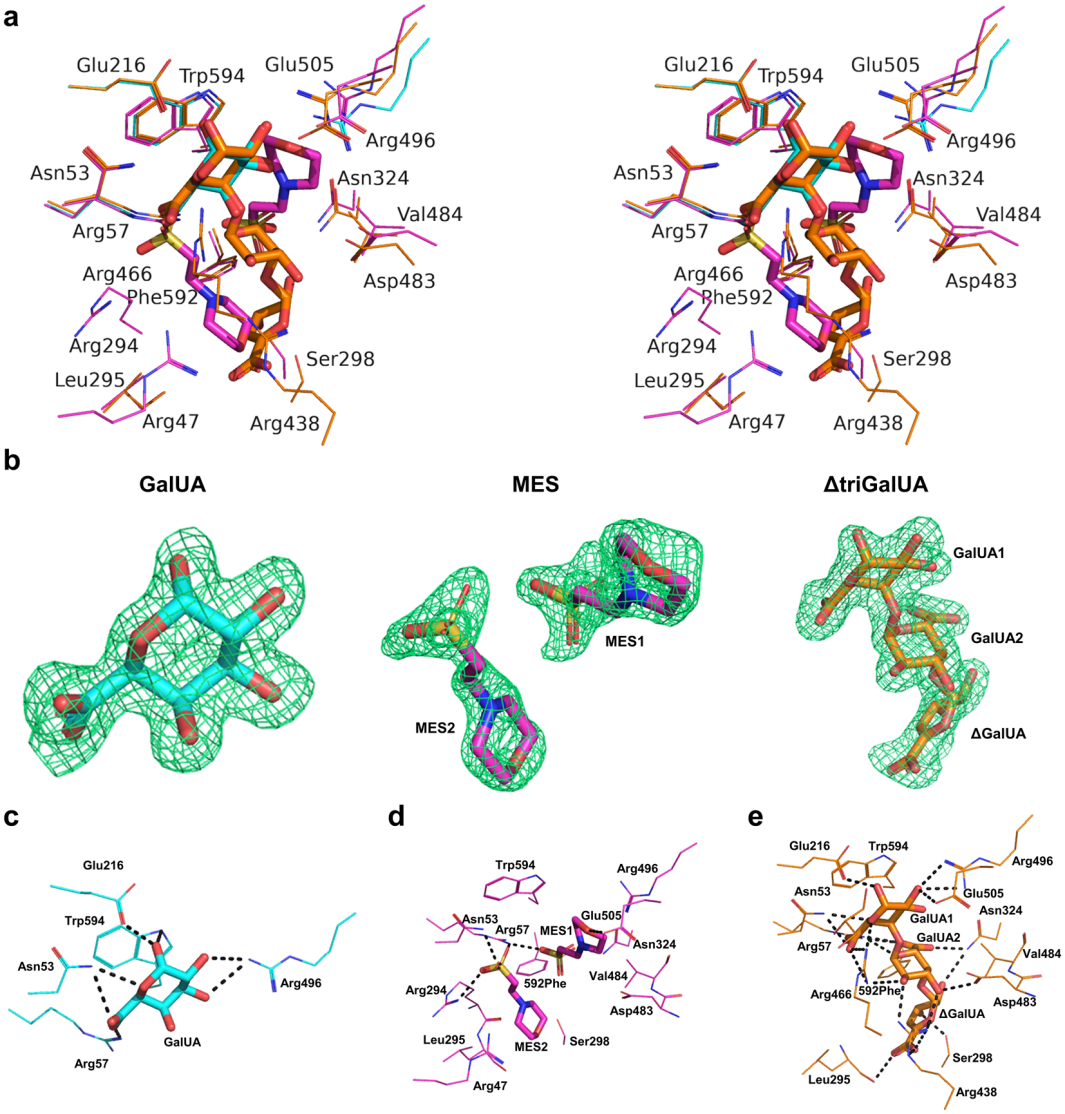
Interactions between SPH1118 and the ligands. Red, blue, and yellow represent oxygen, nitrogen, and sulfur atoms, respectively in (**a**–**e**). (**a**) Residues interacting with GalUA, MES, and ΔtriGalUA via hydrogen bonds and C–C contacts shown in stereo view. Line models show the side chains of the residues interacting with each ligand. Stick models show each ligand (GalUA, MES, and ΔtriGalUA). Cyan, SPH1118/GalUA; magenta, SPH1118/2 × MES; orange, SPH1118/ΔtriGalUA. (**b**) Omit maps of GalUA, MES, and ΔtriGalUA. Electron density maps (*F*_o_–*F*_c_) of each ligand are shown in green mesh contoured with 3.0 σ. (**c**) Residues interacting with GalUA. Dotted lines represent hydrogen bonds. Cyan represents carbon atom. (**d**) Residues interacting with MES. Dotted lines represent hydrogen bonds. Magenta represents carbon atom. (**e**) Residues interacting with ΔtriGalUA. Dotted lines represent hydrogen bonds. Orange represents carbon atom.

Based on the crystal structures complexed with ligands, all ligands were bound to the same area in SPH1118. GalUA bound to SPH1118/GalUA was in the same location as GalUA1 at the reducing end of ΔtriGalUA bound to SPH1118/ΔtriGalUA (Fig. 4a). Conversely, the six-membered ring structures of two MES molecules hardly overlapped with that of ΔtriGalUA. However, the sulfo groups of MES1 and MES2 overlapped with the carboxy groups of GalUA2 (the central GalUA) and GalUA1 of ΔtriGalUA, respectively. The negatively charged sulfo groups interacted with the positively charged basic residues, suggesting that MES could bind to SPH1118 due to the electrical interaction of the sulfo group with the positively charged residues.

Although the pectin oligosaccharide was prepared from RG-I according to the previous report^30^, there were no complexed structures with RG-I disaccharide composed of ΔGalUA and Rha in this study. Surprisingly, the electron density map revealed that the substrate bound to SPH1118 was ΔtriGalUA instead of RG-I disaccharide (Fig. 4b, ΔtriGalUA). This was probably due to the substrate specificity of SPH1118 preferring to bind to acidic sugars. *K*_d_ values of SPH1118 for PG and RG-I were evaluated by UV absorption spectra, resulting in 8.28 ± 1.00 μM and 181 ± 94.0 μM, respectively (Supplementary Table 1), suggesting that SPH1118 preferred acidic sugars because SPH1118 showed a higher affinity for PG consisting of the acidic sugar GalUA than for RG-I containing non-acidic sugars. Arginines, basic amino acid residues, were closely located in the binding pocket of SPH1118/ΔtriGalUA (Arg57, Arg438, Arg466, and Arg496). This is probably one reason for the high affinity of SPH1118 with PG compared to RG-I. The exo-type lyase YesX has been reported to have enzymatic activity for PG^30^. Since the purchased RG-I reagent showed high contents of GalUA (61%), RG-I was considered to contain the RG-II or PG region partly. Therefore, YesX worked on the RG-II or PG region, resulting in the production of ΔtriGalUA. Based on the above, the complex structure with ΔtriGalUA instead of RG-I disaccharide was obtained due to the high affinity of SPH1118 with ΔtriGalUA.

The interactions between the ligand (GalUA, MES, or ΔtriGalUA) and SPH1118 were summarized (Tables 2, 3, 4). There were 13 hydrogen bonds and 26 C–C contacts with GalUA (Fig. 4c). Five residues (Asn53, Arg57, Glu216, Arg496, and Trp594) formed hydrogen bonds and C–C contacts. The nitrogen atom of Arg57 formed a charged hydrogen bond with the carbonyl oxygen atom of GalUA at a distance of 2.7 Å, which may be particularly important for binding to the substrate. Trp594 formed 21 C–C contacts, indicating a strong contribution to the stabilization of the substrate through stacking interactions.

**Table 2:** Interactions in SPH1118/GalUA between SPH1118 and GalUA.

Possible hydrogen bond (< 3.3 Å)					C–C contacts (< 4.5 Å)				
Source sugar	Atom	Target Protein/water	Atom	Distance (Å)	Source sugar	Atom	Target Protein/water	Atom	Distance (Å)
GalUA	O1	Glu216	OE1	2.8	GalUA	C1	Glu216	CD	4.4
	O1	Trp594	NE1	3.2		C1	Trp594	CE2	4.3
	O1	water141	O	3.2		C1	Trp594	CZ2	4.4
	O2	water205	O	2.6		C3	Trp594	CG	3.7
	O2	Arg496	NH2	3.3		C3	Trp594	CD1	3.6
	O2	water141	O	2.8		C3	Trp594	CD2	4.0
	O3	water263	O	3.0		C3	Trp594	CE2	4.1
	O3	Arg496	NH2	3.3		C3	Trp594	CB	4.2
	O3	water247	O	2.9		C4	Trp594	CG	4.0
	O5	Asn53	ND2	3.1		C4	Trp594	CD1	4.4
	OA	water226	O	2.6		C4	Trp594	CD2	4.0
	OA	Arg57	NH1	2.7		C4	Trp594	CE3	4.4
	OB	Asn53	ND2	3.2		C4	Trp594	CB	4.2
						C5	Asn53	CG	4.5
						C5	Trp594	CZ3	4.0
						C5	Trp594	CG	4.2
						C5	Trp594	CD2	3.7
						C5	Trp594	CE2	4.0
						C5	Trp594	CE3	3.7
						C5	Trp594	CZ2	4.3
						C5	Trp594	CH2	4.3
						C6	Asn53	CG	4.2
						C6	Asn53	CB	3.8
						C6	Arg57	CD	4.3
						C6	Trp594	CZ3	4.3
						C6	Trp594	CE3	4.1

**Table 3:** Interactions in SPH1118/2 × MES between SPH1118 and MES.

Possible hydrogen bond (< 3.3 Å)					C–C contacts (< 4.5 Å)				
Source ligand	Atom	Target Protein/water	Atom	Distance (Å)	Source ligand	Atom	Target Protein/water	Atom	Distance (Å)
MES1	O1	Glu505	OE2	3.2	MES1	C2	Trp594	CD1	3.9
	O1S	Arg57	NH1	2.7		C2	Trp594	CG	4.4
	O2S	water60	O	2.9		C3	Trp594	CD1	3.9
	O3S	water158	O	2.3		C3	Trp594	CB	4.1
	O3S	Asn324	ND2	2.8		C3	Trp594	CG	4.2
	N4	water256	O	2.9		C5	Val484	CG2	3.8
MES2	O1	water456	O	3.2		C5	Asp483	CG	4.0
	O1S	Arg294	NH1	2.9		C6	Glu505	CG	3.9
	O2S	Arg57	NH1	2.8		C6	Glu505	CD	3.5
	O2S	water457	O	2.9		C6	Arg496	CZ	4.0
	O3S	Asn53	ND2	3.3		C7	Val484	CG2	4.0
	O3S	water375	O	2.7		C8	Trp594	CB	3.8
	N4	water227	O	3.0	MES2	C3	Arg47	CZ	4.1
						C3	Leu295	CD2	4.0
						C5	Phe592	CZ	4.2
						C6	Ser298	CB	3.9
						C7	Arg294	CG	3.9
						C7	Arg294	CD	4.3

**Table 4:** Interactions in SPH1118/ΔtriGalUA between SPH1118 and ΔtriGalUA.

Possible hydrogen bond (< 3.3 Å)					C–C contacts (< 4.5 Å)				
Source sugar	Atom	Target Protein/water	Atom	Distance (Å)	Source sugar	Atom	Target Protein/water	Atom	Distance (Å)
GalUA1	O1	Glu216	OE1	2.7	GalUA1	C1	Glu216	CD	4.4
	O1	water23	O	3.1		C1	Trp594	CE2	4.4
	O2	Glu505	OE2	2.6		C2	Glu505	CD	4.2
	O2	water23	O	2.9		C3	Trp594	CE2	4.0
	O2	Arg496	NH1	3.1		C3	Trp594	CD1	3.6
	O2	Arg496	NH2	3.3		C3	Trp594	CG	3.6
	O3	water2	O	2.9		C3	Trp594	CD2	3.8
	O3	water91	O	2.9		C3	Trp594	CB	4.1
	O5	Asn53	ND2	3.2		C4	Trp594	CE2	4.4
	O5	Arg466	NH1	2.6		C4	Trp594	CD1	4.4
	O6A	Asn53	ND2	3.2		C4	Trp594	CG	4.0
	O6A	Arg466	NH1	3.0		C4	Trp594	CD2	3.9
	O6A	Arg466	NH2	3.3		C4	Trp594	CE3	4.2
	O6A	water224	O	2.8		C4	Trp594	CB	4.2
	O6B	Arg57	NH1	3.0		C5	Asn53	CG	4.5
	O6B	water324	O	2.7		C5	Trp594	CE2	4.1
GalUA2	O2	Arg466	NH2	3.1		C5	Trp594	CZ2	4.3
	O2	water13	O	2.8		C5	Trp594	CH2	4.3
	O2	Arg438	NH2	2.8		C5	Trp594	CG	4.4
	O3	Asp483	OD1	2.9		C5	Trp594	CD2	3.9
	O3	Arg438	NH1	2.8		C5	Trp594	CE3	3.8
	O5	Arg57	NH1	3.1		C5	Trp594	CZ3	4.0
	O5	water118	O	2.9		C6	Asn53	CG	4.0
	O6A	Arg57	NH1	2.7		C6	Asn53	CB	3.7
	O6A	Arg57	NH2	3.2		C6	Arg466	CZ	4.5
	O6A	water71	O	3.2		C6	Arg57	CD	4.5
	O6B	water71	O	2.9		C6	Trp594	CE3	4.2
	O6B	Asn324	ND2	2.9		C6	Trp594	CZ3	4.2
ΔGalUA	O2	Asn324	ND2	3.2	GalUA2	C2	Arg438	CZ	4.2
	O2	water101	O	2.7		C3	Asp483	CG	4.5
	O3	Ser298	OG	2.7		C4	Val484	CG2	4.1
	O5	Arg438	NH1	3.0		C4	Val484	CG1	4.0
	O5	water109	O	3.3		C6	Trp594	CB	4.5
	O6A	Arg438	NE	2.9		C6	Phe592	CE1	3.9
	O6A	Arg438	NH1	2.4	ΔGalUA	C1	Val484	CG1	3.8
	O6A	water109	O	3.0		C2	Val484	CG1	4.2
	O6B	Leu295	O	2.9		C2	Phe592	CE1	4.4
	O6B	water321	O	2.9		C2	Ser298	CB	4.4
						C3	Phe592	CE1	3.8
						C3	Phe592	CZ	3.8
						C3	Ser298	CB	4.4
						C4	Ser298	CB	4.1
						C5	Arg438	CZ	4.4
						C6	Arg438	CD	4.0
						C6	Arg438	CZ	3.8

There were 13 hydrogen bonds and 18 C–C contacts between the two MES molecules and SPH1118 (Fig. 4d). Five residues (Asn53, Arg57, Arg294, Asn324, and Glu505) formed hydrogen bonds, and ten residues (Arg47, Arg294, Leu295, Ser298, Asp483, Val484, Arg496, Glu505, Phe592, and Trp594) were involved in C–C contacts. The nitrogen atom of Arg57 formed charged hydrogen bonds with the sulfonyl oxygen atoms of the two molecules of MES, and the nitrogen atom of Arg294 formed a charged hydrogen bond with the sulfonyl oxygen atom of one molecule (MES2). Conversely, few stacking interactions via the aromatic ring were observed through Trp594.

Regarding ΔtriGalUA, there were 38 hydrogen bonds and 45 C–C contacts between ΔtriGalUA and SPH1118 (Fig. 4e). Eleven residues (Asn53, Arg57, Glu216, Leu295, Ser298, Asn324, Arg438, Arg466, Asp483, Arg496, and Glu505) interacted with the substrate via hydrogen bonds, and 11 residues (Asn53, Arg57, Glu216, Ser298, Arg438, Arg466, Asp483, Val484, Glu505, Phe592, and Trp594) formed C–C contacts. Focusing on the interactions working on each monosaccharide in ΔtriGalUA, there were 16 hydrogen bonds and 28 C–C contacts found in GalUA1, 12 hydrogen bonds and 6 C–C contacts observed in GalUA2, and 10 hydrogen bonds and 11 C–C contacts found in ΔGalUA. The nitrogen atoms of Arg57 formed three charged hydrogen bonds (GalUA1, one; GalUA2, two), those of Arg438 formed two charged hydrogen bonds with ΔGalUA, and those of Arg466 formed two charged hydrogen bonds with GalUA1. Regarding the number of hydrogen bonds in each amino acid residue, Arg57, Arg438, and Arg466 formed four, five, and four hydrogen bonds, respectively, indicating that these residues were important among the binding sites. Moreover, Trp594 formed 22 C–C contacts (GalUA1, twenty-one; GalUA2, one), which were considered significantly important for stabilizing the substrate via stacking interactions. SPH1118 was bound to ΔtriGalUA through many hydrogen bonds and salt bridges (Arg57, Arg438, and Arg466) and stacking interactions (Trp594). This also suggested that GalUA1 in ΔtriGalUA was particularly bound to SPH1118 strongly.

### Overall and conformational changes of SPH1118

**Figure 5:**
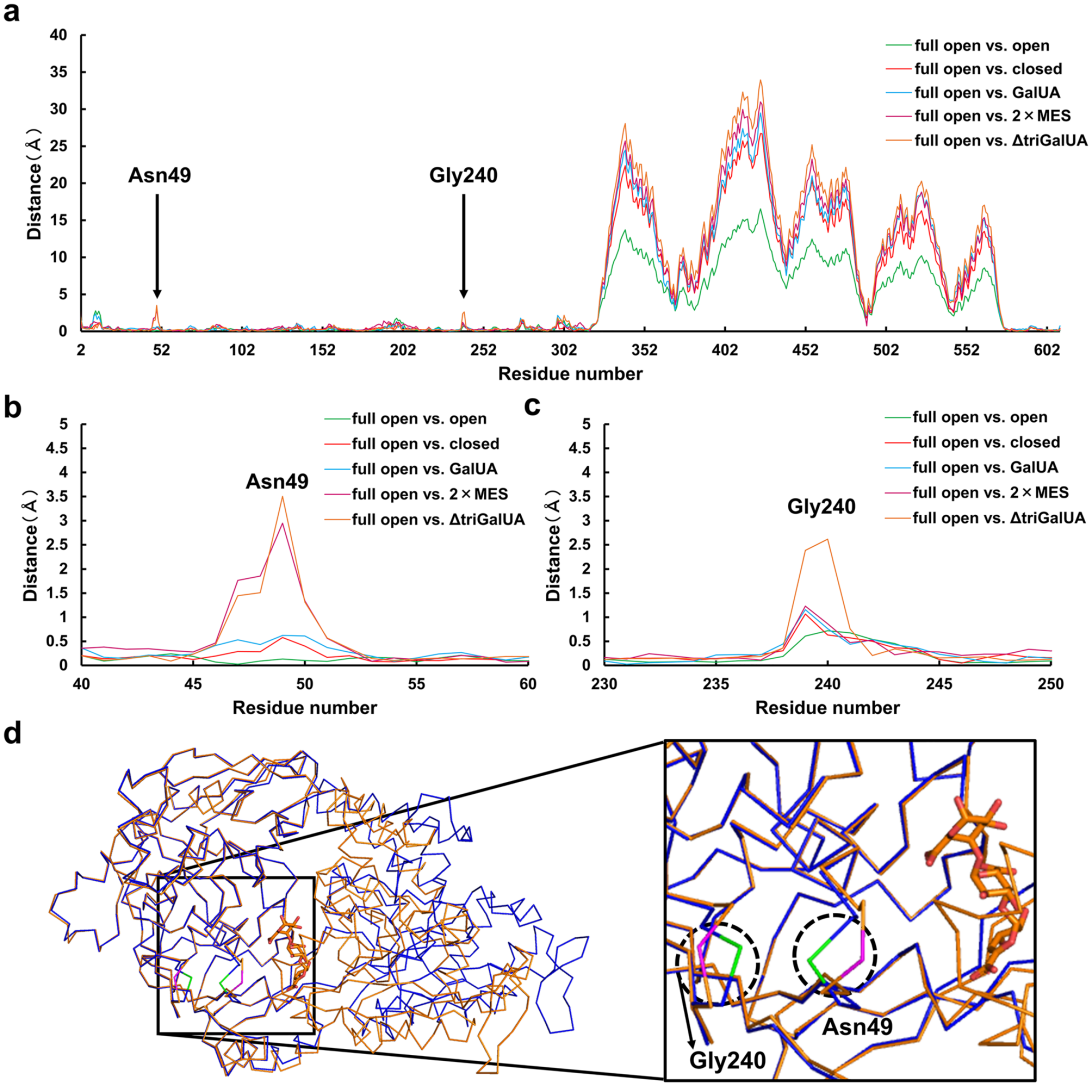
Conformational change in each residue of SPH1118. (**a**) Distance of the Cα at each residue (2–610) relative to that of SPH1118 (full open form) in the superimposed structure at N terminal domain. Green, SPH1118 (open form) vs. SPH1118 (full open form); red, SPH1118 (closed form) vs. SPH1118 (full open form); cyan, SPH1118/GalUA vs. SPH1118 (full open form); magenta, SPH1118/2 × MES vs. SPH1118 (full open form); orange, SPH1118/ΔtriGalUA vs. SPH1118 (full open form). (**b**) Distance of the Cα at each residue (40–60) relative to SPH1118 (full open form). (**c**) Distance of the Cα at each residue (230–250) relative to SPH1118 (full open form). (**d**) Local conformational changes in SPH1118. Ribbon and stick models show the main chain of SPH1118 and ΔtriGalUA, respectively. Blue, SPH1118 (full open form); orange, SPH1118/ΔtriGalUA; green, Asn49 and Gly240 in SPH1118 (full open form): magenta, Asn49 and Gly240 in SPH1118/ΔtriGalUA.

As mentioned above, SPH1118 could adopt various conformations in response to the substrate size. The distance among the Cα at each residue relative to SPH1118 (full open form) was calculated using the superimposed structures based on the N terminal domain to investigate the regions responsible for the flexibility of such conformational changes (Fig. 5a). As a result, the C terminal domain (residues 323–572) exhibited similar behavior, although there was a difference in the degree of closure among conformations. On the other hand, there were remarkable variations of the specific residues near the substrate-binding site in the N terminal domain of SPH1118/ΔtriGalUA: Asn49 (Fig. 5b) and Gly240 (Fig. 5c). Asn49 in SPH1118/2 × MES and SPH1118/ΔtriGalUA was found to undergo the significant conformational changes as seen in Gly240 in SPH1118/ΔtriGalUA. The main chain of Asn49 in SPH1118 (full open form) was located on the opposite side of the binding pocket (Fig. 5d, green), while that of Asn49 in SPH1118/ΔtriGalUA was located in the direction of the binding pocket (Fig. 5d, magenta). Conversely, Gly240 of SPH1118 (full open form) was in the direction of binding pocket, whereas Gly240 of the SPH1118/ΔtriGalUA moved to the opposite side. In these residues, Gly240 was included in the extra domain. There were few direct interactions between these residues and the substrate. However, little variation of these residues in SPH1118/GalUA was observed. This suggested that such local conformational changes were responsible for enabling SPH1118 to bind to oligosaccharides and polysaccharides.

## Discussion

SPH1118 contributes to pectin chemotaxis and assimilation in strain A1. In this study, six variations of the crystal structures of SPH1118 were determined. These structures revealed the recognition mechanism of the substrate and conformational changes in response to the substrate size. In contrast, SPH1118 was annotated as an oligopeptide-binding protein based on the primary structure. Gene homology analysis predicted that genes were encoding ABC transporters for oligopeptides in the vicinity of *sph1118*. A DSF assay was conducted to examine whether SPH1118 was bound to oligopeptides. SPH1118 was hardly bound to yeast extract or tryptone (Supplementary Fig. 2b, c). Yeast extract is a digestive product of yeast cells degraded by proteases and contains more than 4000 oligopeptides^31^. Tryptone is a product of the hydrolysis of casein with trypsin. *T*_m_ values of SPH1118 in the absence and presence of 1 mg/ml yeast extract were 52.7 and 52.5 °C, respectively. *T*_m_ values of SPH1118 in the absence and presence of 1 mg/ml tryptone were 52.7 and 52.8 °C, respectively. These results suggested that SPH1118 specifically recognized pectin rather than oligopeptides. The structural features of SPH1118 explained the reason for this substrate specificity. SPH1118 showed 37% identity in the primary structure with the oligopeptide-binding protein OppA (PDB-ID: 5IPW) from hyperthermophilic gram-negative bacterium *Thermotoga maritima*. OppA was used as a model for molecular replacement in the structural determination of SPH1118 in this study. In previous report, there was no complex structure of OppA with oligopeptide^32^. The structural determinants responsible for substrate recognition were explored by comparing SPH1118 and OppA. RMSD of SPH1118 to OppA was calculated to be 1.6 Å, revealing that SPH1118 was structurally similar to OppA. There was a difference in electrostatic potential. The interdomain cleft of SPH1118 was positively charged, while that of OppA was negatively charged (Fig. 2c, Supplementary Fig. 3a). Since pectin is negatively charged, SPH1118 was considered to bind to pectin via electrical charge interaction. In OppA, only a few residues are conserved in the substrate-binding residues of SPH1118 (Supplementary Fig. 4). In particular, Arg57, Arg438, and Trp594 are hardly conserved in OppA. Two arginines (Arg57 and Arg438) and Trp594 stabilized the substrate through salt bridges and stacking interactions, respectively, suggesting that these residues were particularly important for binding to the substrate. The differences in these residues were probably responsible for the substrate specificity of SPH1118 for pectin rather than oligopeptides.

OppA is found to have a large cleft between domains, which allows it to accommodate long oligopeptides as long as 20 amino acid residues^32^. Thus, the cleft volumes of SPH1118 and OppA were calculated using *ProFunc* program^33^. As a result, the cleft volumes of SPH1118 and OppA were 9088 Å^3^ and 10,638 Å^3^, respectively, indicating that both proteins had large clefts. TogB of *D. dadantii* is functionally similar to SPH1118 because TogB is involved in the expression of pectin oligosaccharides chemotaxis and transport^21^. However, the tertiary structure of *D. dadantii* TogB remains to be clarified. The structure of gram-negative bacterium *Yersinia enterocolitica* TogB complexed with pectin oligosaccharides (PDB-ID: 2UVJ) has been determined^34^. *Y. enterocolitica* is able to assimilate pectin, as seen in *D. dadantii*. TogB of *Y. enterocolitica* exhibits a high affinity with pectin oligosaccharides. *Y. enterocolitica* TogB shows high sequence identity (79%) with *D. dadantii* TogB, while exhibits little sequence identity (12%) with SPH1118. The surface charge and cleft volume of TogB were calculated. The substrate-binding pocket was positively charged, as seen in SPH1118, while the cleft volume of TogB was 3564 Å^3^ (Supplementary Fig. 3b). The cleft volume of SPH1118 was about 2.5 times as large as that of TogB. Although SPH1118 could bind to PG, TogB is hardly bound to PG^34^. This was considered to be due to the differences in the cleft volumes of both proteins. The large and positively charged cleft of SPH1118 allowed the binding to acidic polysaccharide pectin. Docking simulation using *SwissDock* program^35^ revealed that SPH1118 (open form) could accommodate heptagalacturonic acid used as a part of polysaccharide model in the large cleft (Supplementary Fig. 3c, Movie 1). This result suggested that the structure of SPH1118 was suitable for binding to the polysaccharide.

SPH1118 was crystallized in the presence of some substrates to determine the crystal structures with six different conformations. Interestingly, SPH1118 showed three conformations [SPH1118 (open form), SPH1118 (full open form), and SPH1118 (closed form)] even in the substrate-free state. These conformations were probably present in equilibrium in solution. To confirm whether SPH1118 shows conformational changes under physiological conditions, observation of the state of SPH1118 in solution is necessary using X-ray solution scattering. The crystal structures of SPH1118 (full open form) and SPH1118 (closed form) were obtained only under crystallization conditions in the presence of sodium PG. Conversely, the crystal structure of SPH1118 (open form) was obtained under several different conditions other than those shown in Supplementary Table 2, indicating that SPH1118 (open form) was likely to have a more stable conformation than any other substrate-free conformation. SPH1118 (full open form) opened the domains more than SPH1118 (open form), thereby increasing the surface area exposed to the solvent and possibly accommodating the polysaccharide more efficiently. There are a few examples of proteins whose domains are closed in the substrate-free state, as observed in SPH1118 (closed form). The galactose/glucose-binding protein GGBP and the choline/acetylcholine-binding protein ChoX have closed conformation in the substrate-free state^36,37^. There is little structural difference between the closed forms in the substrate-free state and the substrate-bound state. SBPs bound to substrates enhance the ATP activity of the ABC transporter more strongly than non-bound SBPs^38^. Further studies are necessary to elucidate the physiological significance of SBPs adopting the closed conformation in the absence of the substrate.

SPH1118 showed large conformational changes among the substrate-free states and had an interesting feature of inducing conformational changes even in the substrate-bound state. As the molecular weight of the substrate increased, the domain of SPH1118 was more closed. In general, the conformations of the substrate-bound structures of SBPs are stabilized in the closed states and rarely show different degrees of closure in response to the bound substrate^39^. For example, the alginate polysaccharide-binding proteins AlgQ1 and AlgQ2 in strain A1 hardly exhibit any conformational change in response to the length of the alginate oligosaccharide^40,41^. Regarding TogB, which is similar to SPH1118 in function, the complexed structures with digalacturonic acid, unsaturated digalacturonic acid, and trigalacturonic acid (triGalUA) have been reported. However, all of these structures show few significant structural differences^34^. To the best of our knowledge, this is the first example of SBP causing such conformational change in response to the substrate size. The substrate-binding site of SPH1118/ΔtriGalUA consisted of three subsites (GalUA1, subsite 1; GalUA2, subsite 2; ΔGalUA, subsite 3). GalUA of SPH1118/GalUA bound to subsite 1, suggesting that subsite 1 was particularly important for binding of the substrate. The structural difference between SPH1118/GalUA and SPH1118/ΔtriGalUA was probably explained by the presence or absence of the substrate in subsites 2 and 3. GalUA1 of SPH1118/ΔtriGalUA was superimposed on GalUA of SPH1118/GalUA to investigate the interactions between the substrate and amino acids located in subsites 2 and 3. As a result, five specific residues (Arg57, Asn324, Arg438, Arg466, and Asp483) of SPH1118/ΔtriGalUA were found to move toward the substrate, resulting in the formation of new hydrogen bonds in subsites 2 and 3 (Supplementary Fig. 5, Supplementary Table 3). This suggested that these movements resulted in the overall conformational change of the N and C terminal domains to be stable state. Although there were no crystal structures of SPH1118 bound to polysaccharide pectin or PG in this study, the domain of the complex structures with pectin or PG was likely to be as closed as that of SPH1118/ΔtriGalUA or more. Since the MCP and the ABC transporter of pectin in strain A1 remain to be clarified, further studies are required to clarify the physiological significance of SPH1118 showing different conformations among the substrate-bound states.

**Figure 6:**
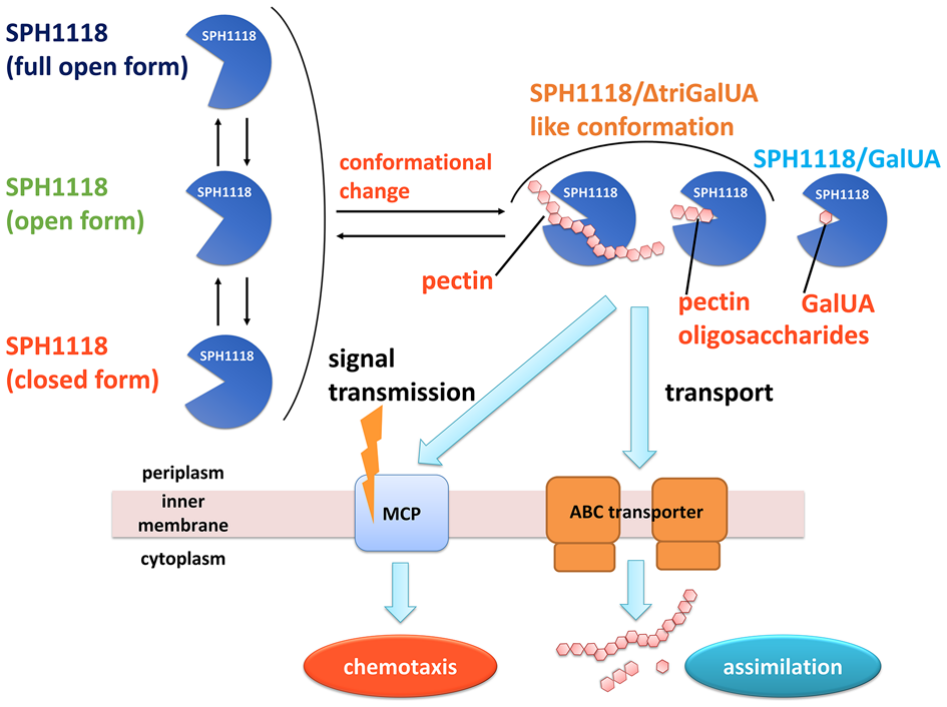
The model of SPH1118-mediated pectin chemotaxis and assimilation in strain A1. Substrate-free SPH1118 has three conformations: SPH1118 (open form), SPH1118 (full open form), and SPH1118 (closed form). Binding of SPH1118 to the substrate induces a further conformational change to interact with the MCP-like receptors to express chemotaxis toward pectin and also with the ABC transporters to transport the substrate.

Although the mechanism of pectin transport and degradation in strain A1 remains to be clarified, this study provided further insight into the functioning mechanism of SPH1118 (Fig. 6). According to the topological analysis based on the *PSORTb* program^42^, SPH1118 was predicted to locate in the periplasm. In the absence of the substrate, SPH1118 in the periplasm adopts three conformations in equilibrium as follows: SPH1118 (open form), SPH1118 (full open form), and SPH1118 (closed form). Upon binding to the substrate, SPH1118 is predicted to undergo the conformational change and show SPH1118/ΔtriGalUA-like conformation. Subsequently, SPH1118 might be able to interact with both MCPs and ABC transporters, which would trigger pectin chemotaxis expression and its assimilation.

In conclusion, this study is the first report to clarify the polysaccharide recognition mechanism and conformational changes of the bifunctional SBP involved in polysaccharide chemotaxis and assimilation.

## Methods

### Materials

Pectin from citrus fruits and SYPRO Orange were purchased from Sigma-Aldrich. GalUA was purchased from Fujifilm Wako Pure Chemical. PG and RG-I were purchased from Megazyme. All other chemicals used in this study were analytical-grade and commercially available.

### Bacterial strains and culture

*E. coli* BL21(DE3)/pET21b-*sph1118*, which was already constructed^19^, was used for overexpression of the recombinant protein. *E. coli* cells were cultured aerobically at 37 °C in LB medium [1% (w/v) tryptone, 0.5% yeast extract, and 1% sodium chloride (pH 7.2)] with sodium ampicillin at a concentration of 0.1 mg/ml.

### Purification of SPH1118

SPH1118 was purified as reported previously^19^ with slight modifications. The overexpression system of SPH1118 has been previously constructed in *E. coli* cells^19^. SPH1118 was purified from the *E. coli* cell extract using TALON histidine-tag fusion protein purification resin (Clontech) column, HiLoad 16/10 Phenyl Sepharose (GE Healthcare Life Sciences), and HiLoad 16/600 Superdex 200 pg (GE Healthcare Life Sciences). The eluted fractions were subjected to SDS-PAGE to confirm the high purity of SPH1118. The concentration of SPH1118 was determined by measuring *A*_280_ based on the molar absorption coefficient of 122,730 (M^-1^ cm^-1^) calculated using the *Expasy ProtParam* tool^43^.

### Preparation of pectin oligosaccharide

Pectin oligosaccharide was prepared from RG-I, as previously reported^30^. Briefly, acid hydrolysis treatment was performed on the side chains of RG-I. Cell lysates of YesX-expressing recombinant *E. coli*^30^ were added to 2% RG-I main chain solution containing 20 mM MnCl_2_ and 50 mM tris-hydroxymethyl aminomethane-hydrochloric acid (Tris–HCl) buffer (pH 7.5) followed by enzymatic reaction at 30 °C for 24 h. The mixture was boiled for 10 min to inactivate the enzyme. After centrifugation, the supernatant was subjected to Centriprep Centrifugal Filter Unit (Merck Millipore) to collect the products with molecular weight of less than 3,000. The collected products were purified using Superdex Peptide 10/300 GL (GE Healthcare Life Sciences) and Bio-Gel P2 column (Bio-Rad). Elution was performed with distilled water. The collected fractions were subjected to TLC to confirm the purification of pectin oligosaccharides. The pectin oligosaccharide prepared in this study was found to include ΔtriGalUA (ΔGalUA-GalUA-GalUA) as a result of X-ray crystallography of the SPH1118-substrate complex.

### UV absorption spectra assay

The affinity of SPH1118 with the substrates was calculated by measuring the change in *A*_280_ with various substrate concentrations using UV–VIS spectrophotometer UV-2600 (Shimadzu), as previously reported^19^. The changes in absorbance derived from the binding of SPH1118 to the substrates were measured in the wavelength range of 200–400 nm. The difference in *A*_280_ before and after adding the substrates was defined as *ΔA*_280_. The data of *ΔA*_280_ as a function of substrate concentration were fit to the Langmuir equation^25^ to calculate *K*_d_ values.

### DSF assay

The thermal stability of SPH1118 in the absence or presence of the ligands was measured by DSF^26^. SYPRO Orange was used as a fluorescent substance. This substance binds to the hydrophobic region of the protein and emits fluorescence. Except for the ligands, the solution contained 2.9 µM SPH1118, 100-fold diluted SYPRO Orange, and 100 mM Tris–HCl (pH 7.5). The solution was heated from 25 to 95 °C in 0.5 °C steps every 10 s, and the relative fluorescence unit (RFU) was measured at each temperature. The temperature showing the minimal–d(RFU)/dT value was defined as *T*_m_.

### X-ray crystallography

Crystals of SPH1118 were prepared by sitting drop vapor diffusion using 96-well plates. Crystallization was performed by adding 1 μl of reservoir solution to 1 μl of purified SPH1118 solution (20.4–23.9 mg/ml) followed by incubation at 20 °C. The crystallization conditions for each conformation are shown in Supplementary Table 2.

The crystals were flash-cooled in a nitrogen gas stream at –173 °C. The diffraction data were collected by irradiating the crystals with X-ray at 1.0 Å wavelength at the BL-26B1 beamline in SPring-8 (Hyogo, Japan). The *XDS* program^44^ was used to process the diffraction data. Each crystal structure was determined through molecular replacement with the *Molrep* program^45^ in the *CCP4interface* (*CCP4i*) package. The OppA (PDB-ID:5IPW) structure was used as a model for a molecular replacement to determine the structure of SPH1118/2 × MES^32^. The structures of SPH1118 with other conformations were determined by molecular replacement using the structure of SPH1118/2 × MES as a reference model. The *Refmac5*^45^ and *phenix.refine* programs^46^ were used for structural refinement. For each refinement, the models were manually adjusted using the *WinCoot* program^47^. Images (Figs. 2a–d, 3a,b, 4a–e, and 5d, and Supplementary Figs. 3a-c, 5, and 6) of protein structures were prepared using the *PyMOL* molecular graphics system (version 2.4.1) (Schrödinger, USA). The rotation angle of the domain was evaluated using *RotationAxis* (draw_rotation_axis.py; python script by Pablo Guardado Calvo and available from PyMOLWiki, https://pymolwiki.org).

## Supplementary Information

Supplementary Information 1.

Supplementary Video 1.

## Data Availability

The crystallographic diffraction data and atomic coordinates generated in this study have been deposited in the world-wide Protein Data Bank (wwPDB) under accession codes 7VEQ, 7VER, 7VET, 7VEU, 7VEV, and 7VEW. The other X-ray crystal structure coordinates used in this study are available from in wwPDB under accession codes 5IPW and 2UVJ.
