# Supplementary material for: Substrate size-dependent conformational changes of bacterial pectin-binding protein crucial for chemotaxis and assimilation
Source: Sci Rep. 2022 Jul 25;12:12653. doi: 10.1038/s41598-022-16540-5 (PMC9314435; doi:10.1038/s41598-022-16540-5)
Supplement: Supplementary file 1 — Supplementary Information 1. [file 41598_2022_16540_MOESM1_ESM.pdf]

Supplementary Information

Substrate size-dependent conformational changes of bacterial pectin-binding protein crucial for chemotaxis and assimilation

Kotaro Anamizu[1], Ryuichi Takase[1,2], Mamoru Hio[2], Daisuke Watanabe[1,2], Bunzo Mikami[3,4], and Wataru Hashimoto[1,2*]

[1]Laboratory of Basic and Applied Molecular Biotechnology, Division of Food Science and Biotechnology, Graduate School of Agriculture, Kyoto University, Uji, Kyoto 611-0011, Japan
[2]Laboratory of Basic and Applied Molecular Biotechnology, Department of Food Science and Biotechnology, Faculty of Agriculture, Kyoto University, Uji, Kyoto 611-0011, Japan
[3]Laboratory of Metabolic Sciences of Forest Plants and Microorganisms, Research Institute for Sustainable Humanosphere, Kyoto University, Uji, Kyoto 611-0011, Japan
[4]Laboratory of Structural Energy Bioscience, Institute of Advanced Energy, Kyoto University, Uji, Kyoto 611-0011, Japan.

Correspondence and request for materials should be addressed to W.H. (e-mail: hashimoto.wataru.8c@kyoto-u.ac.jp).

Supplementary Table 1 K_d values for substrates of SPH1118 determined by the UV absorption spectra.

Supplementary Table 2 Crystallization conditions for SPH1118.

Supplementary Table 3 Newly formed hydrogen bonds in subsites 2 and 3 caused by the conformational change from SPH1118/GalUA to SPH1118/ΔtriGalUA.

Supplementary Figure 1 Purification of SPH1118 and pectin oligosaccharides.

Supplementary Figure 2 Binding assay of SPH1118 to MES, yeast extract, and tryptone using DSF.

Supplementary Figure 3 Surface charge of OppA, TogB, and SPH1118.

Supplementary Figure 4 Sequence alignment of SPH1118 and OppA.

Supplementary Figure 5 Newly formed hydrogen bonds in subsites 2 and 3 caused by the conformational change from SPH1118/GalUA to SPH1118/ΔtriGalUA.

Supplementary Movie 1 Conformational changes of SPH1118.

Supplementary Table 1 K_d values for substrates of SPH1118 determined by the UV absorption spectra.

**Substrate**	**K_d (μM)**
Pectin	9.28 ± 1.12
PG	8.28 ± 1.00
RG-I	181 ± 94.0
ΔtriGalUA	96.2 ± 10.4

Supplementary Table 2 Crystallization conditions for SPH1118.

Conformation	SPH1118 concentration	Crystallization conditions	Cryoprotectants
SPH1118<br>(open form)	20.4 mg/ml	2.0 M Ammonium dihydrogen phosphate<br>0.1 M Tris-HCl (pH 8.5)<br>2.0 mM ΔTriGalUA	26% Glycerol
SPH1118<br>(full open form)	23.9 mg/ml	25% Polyethylene glycol monomethyl ether 550<br>0.1 M MES (pH 6.5)<br>0.01 M Zinc sulfate<br>20.9 μM Sodium PG	23% Glycerol
SPH1118<br>(closed form)	23.9 mg/ml	20% Polyethylene glycol 3,350<br>0.2 M Ammonium formate<br>20.9 μM Sodium PG	23% Glycerol
SPH1118/GalUA	22.7 mg/ml	40% Polyethylene glycol 600<br>0.1 M Citrate (pH 5.5)<br>50 mM Sodium GalUA	None
SPH1118/2 × MES	21.2 mg/ml	20% Polyethylene glycol 6,000<br>0.2 M Calcium chloride<br>0.1 M MES (pH 6.0)	26% Glycerol
SPH1118/ΔtriGalUA	22.7 mg/ml	22% Polyethylene glycol 3,000<br>0.15 M Potassium sodium tartrate<br>0.1 M 4-(2-Hydroxyethyl)-1-piperazineethanesulfonic acid (pH 7.2)<br>23.4 mM ΔTriGalUA	20% Glycerol

Supplementary Table 3 Newly formed hydrogen bonds in subsites 2 and 3 caused by the conformational change from SPH1118/GalUA to SPH1118/ΔtriGalUA.

**Possible hydrogen bond (<3.3 Å)**					
**Source Protein**	**Atom**	**Target Sugar**	**Atom**	**Distance (Å) (SPH1118/GalUA)**	**Distance (Å) (SPH1118/ΔtriGalUA)**
Arg57	NH1	GalUA2	O5	3.4	3.1
	NH2	GalUA2	O6A	3.8	3.2
Asn324	ND2	ΔGalUA	O2	3.7	3.2
Arg438	NH2	GalUA2	O2	4.2	2.8
	NH1	GalUA2	O3	4.8	2.8
	NH1	ΔGalUA	O5	4.2	3.0
Arg466	NH2	GalUA2	O2	5.1	3.1
Asp483	OD1	GalUA2	O3	3.9	2.9

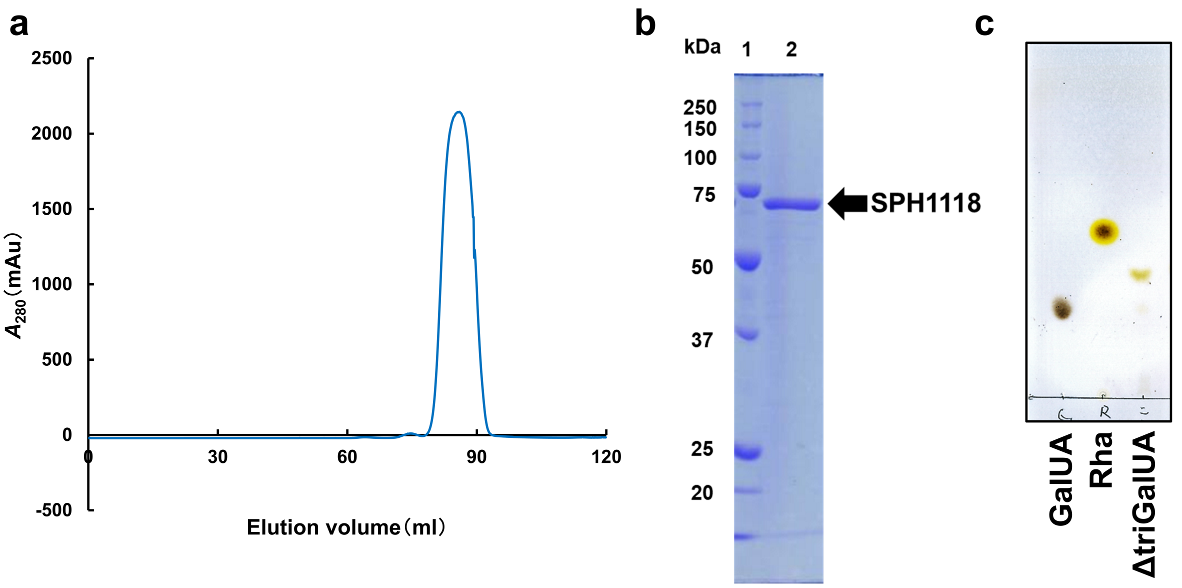

Supplementary Figure 1 Purification of SPH1118 and pectin oligosaccharides. (**a**) Elution profile of the recombinant SPH1118 from gel filtration column (HiLoad 16/600 Superdex 200 pg) chromatography. (**b**) SDS-PAGE profile of the recombinant SPH1118. Lane 1, marker; Lane 2, purified SPH1118. This electrophoretic profile is not an image cropped from different parts of the same gel or different gels. The original full-length gel is shown below. (**c**) TLC analysis of pectin oligosaccharide (ΔtriGalUA). Galacturonic acid (GalUA) and rhamnose (Rha) were used for comparison. The profile of TLC plate is not an image cropped from different parts of the same plate or different plates.

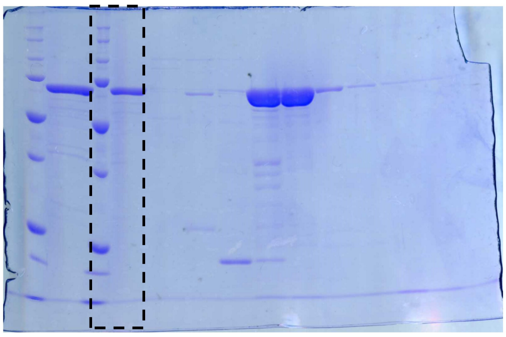

Original SDS-PAGE profile of recombinant SPH1118 (Supplementary Figure 1b). Inside of dotted box corresponds to Supplementary Figure 1b.

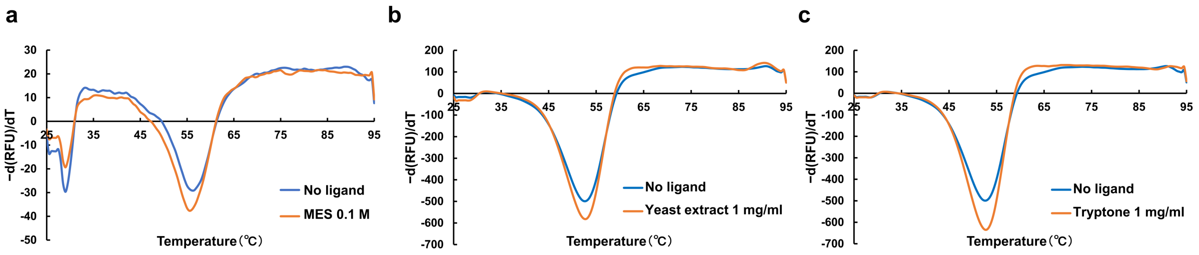

Supplementary Figure 2 Binding assay of SPH1118 to MES, yeast extract, and tryptone using DSF. (**a**) The negative derivative curve plots obtained from the fluorescence profiles of SPH1118 in the absence (blue) and presence of 0.1 M MES (orange). (**b**) The negative derivative curve plots of SPH1118 in the absence (blue) and presence of 1 mg/ml yeast extract (orange). (**c**) The negative derivative curve plots of SPH1118 in the absence (blue) and presence of 1 mg/ml tryptone (orange).

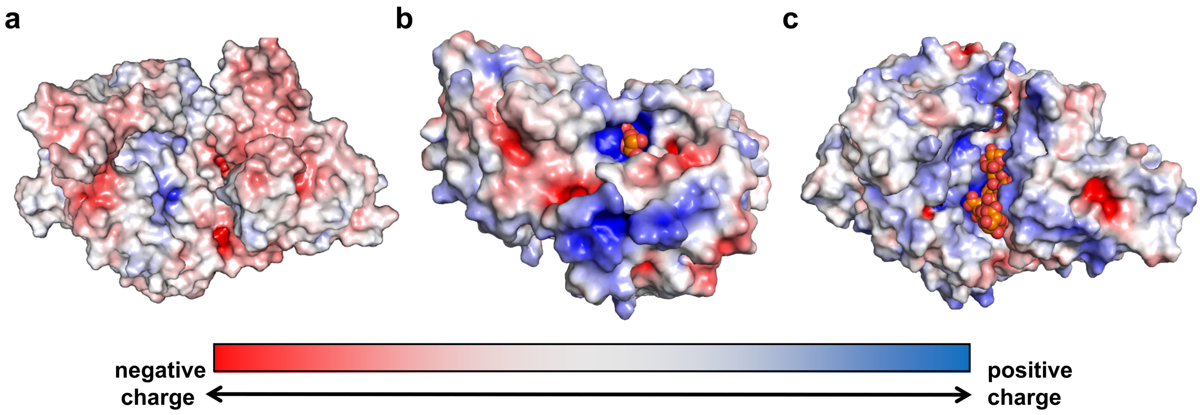

Supplementary Figure 3 Surface charge of OppA, TogB, and SPH1118. Blue and red surfaces refer to positively and negatively charged areas, respectively. (**a**) Surface charge profile of OppA (PDB-ID: 5IPW) at pH 7.0. (**b**) Surface charge profile of TogB complex with trigalacturonic acid (triGalUA) (PDB-ID: 2UVJ) at pH 7.0. Ball model shows triGalUA. Orange and red represent carbon and oxygen atoms, respectively. (**c**) Surface charge profile of SPH1118 complex with heptagalacturonic acid at pH 7.0. This structure was revealed by *SwissDock* program using SPH1118 (open form) and heptagalacturonic acid. Orange ball model shows heptagalacturonic acid. Red represents an oxygen atom.

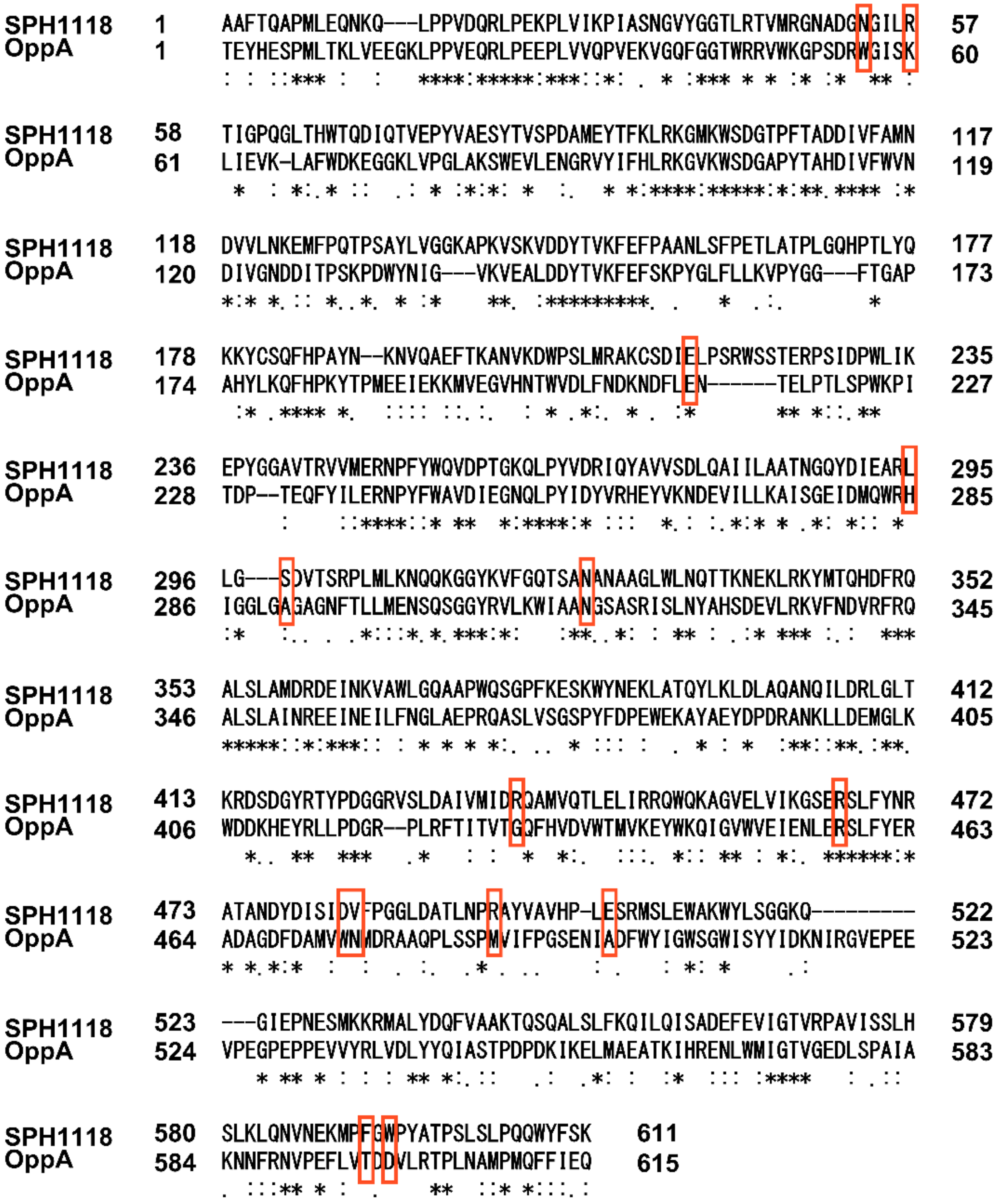

Supplementary Figure 4 Sequence alignment of SPH1118 and OppA. Signal sequence was not included in SPH1118. Red boxes show the residues of SPH1118 interacting with ΔtriGalUA.

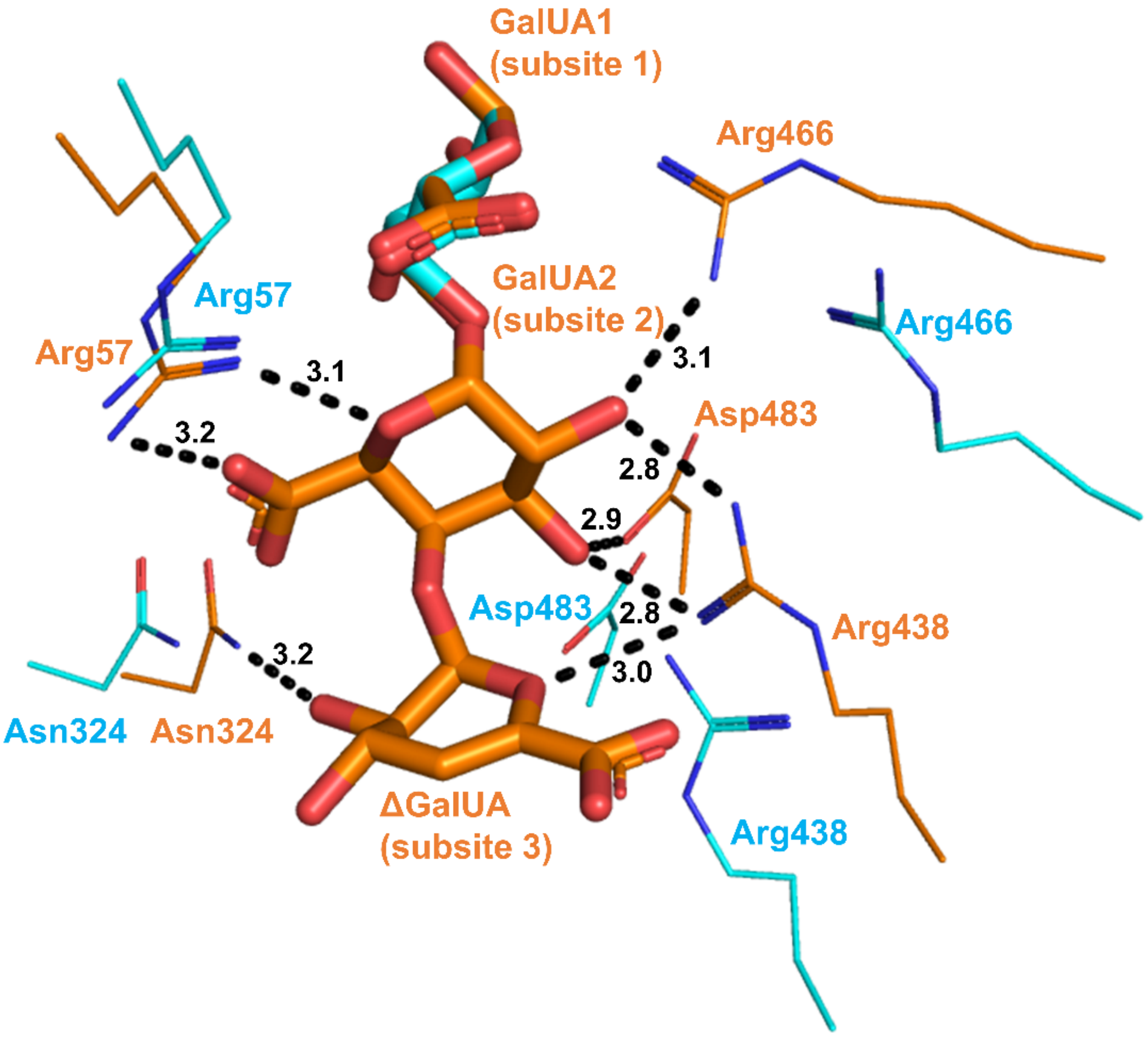

Supplementary Figure 5 Newly formed hydrogen bonds in subsites 2 and 3 caused by the conformational change from SPH1118/GalUA to SPH1118/ΔtriGalUA. Cyan and orange represent carbon atoms of SPH1118/GalUA and SPH1118/ΔtriGalUA, respectively. Stick models show GalUA and ΔtriGalUA. Red and blue represent oxygen and nitrogen atoms, respectively. Dotted lines show hydrogen bonds formed in subsites 2 and 3 with labeled distances (Å).

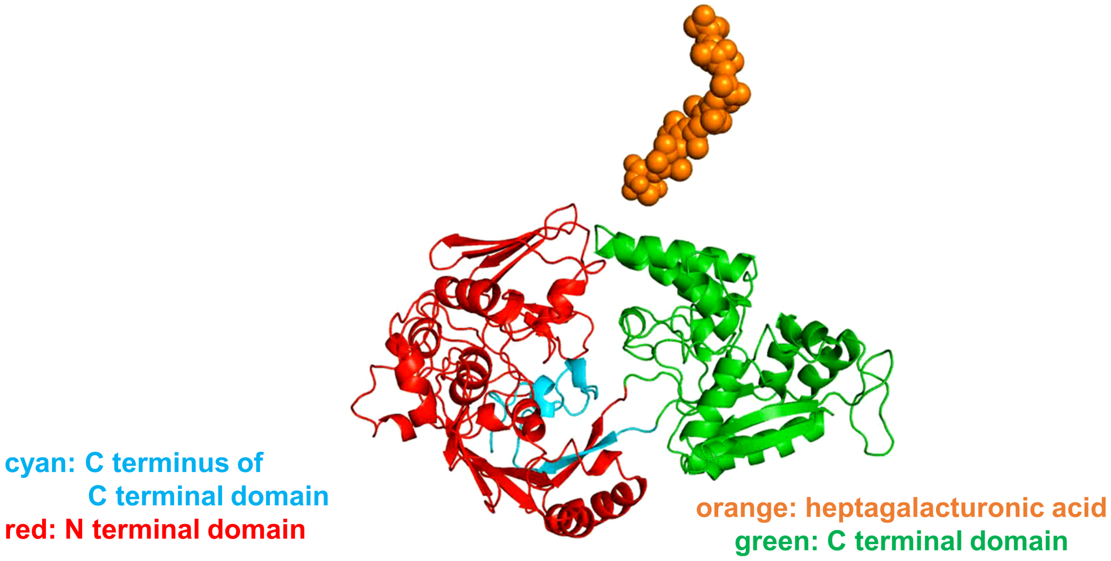

Supplementary Movie 1 Conformational changes of SPH1118. There is one video segment with a total duration of 20 seconds.
